# Dorsal Arachnoid Web: A Rare Cause of Myelopathy

**DOI:** 10.5334/jbsr.2592

**Published:** 2021-12-30

**Authors:** Arvy Buttiens, Bart Feyen, Sven Dekeyzer

**Affiliations:** 1 Antwerp University Hospital, BE

**Keywords:** Spine, myelopathy, arachnoid, web, MRI, CT myelography

## Abstract

**Teaching point:** The scalpel sign is pathognomonic for a dorsal arachnoid web, a rare clinical-radiologic entity that can cause myelopathy.

## Case Description

A 43-year-old Caucasian male presented with interscapular pain and sensory deficits in dermatome Th6-Th7. The pain typically occurred after flexion or rotation of the spine and had gradually worsened over the past year.

**Figure 1:**
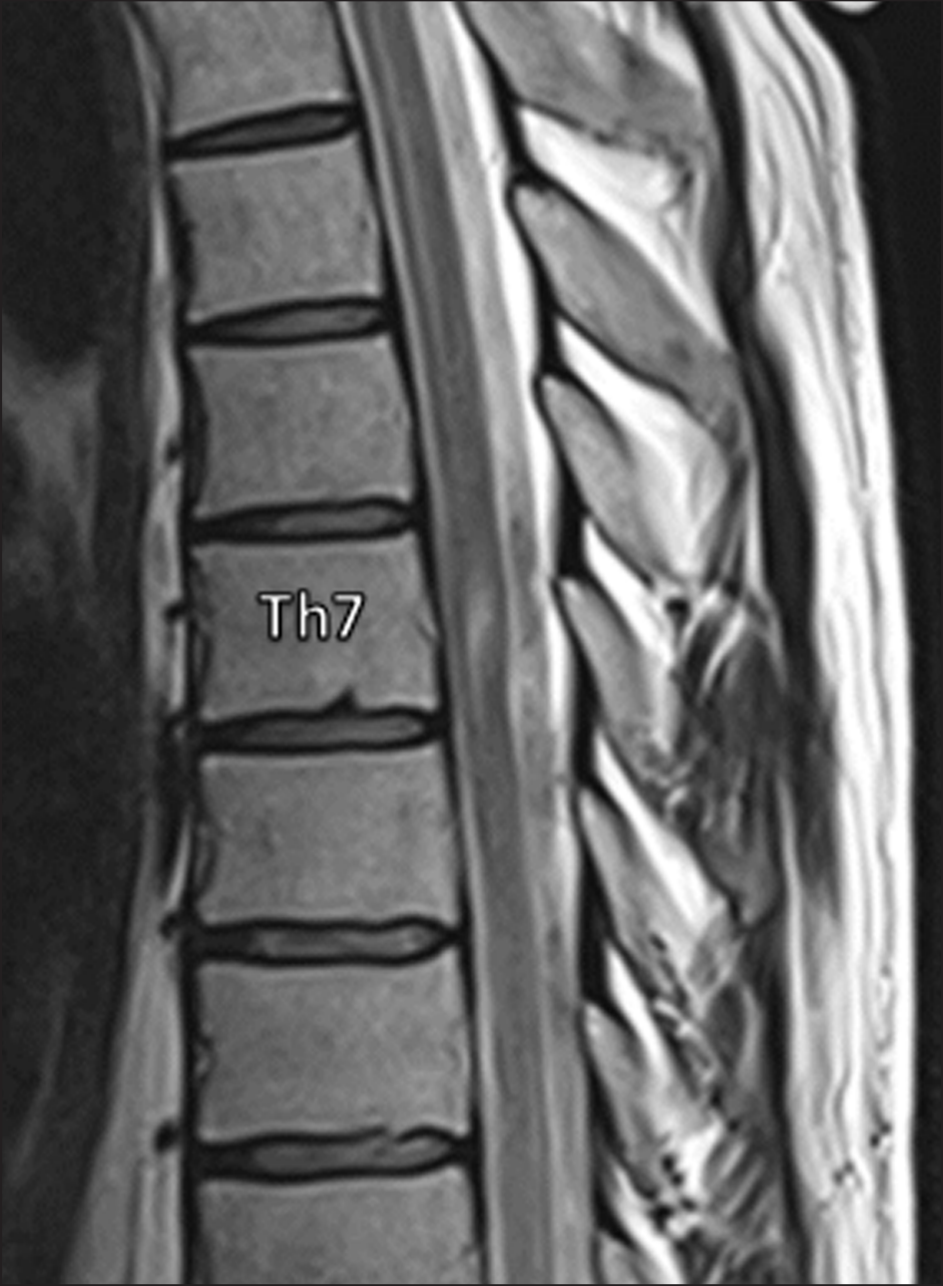


**Figure 2:**
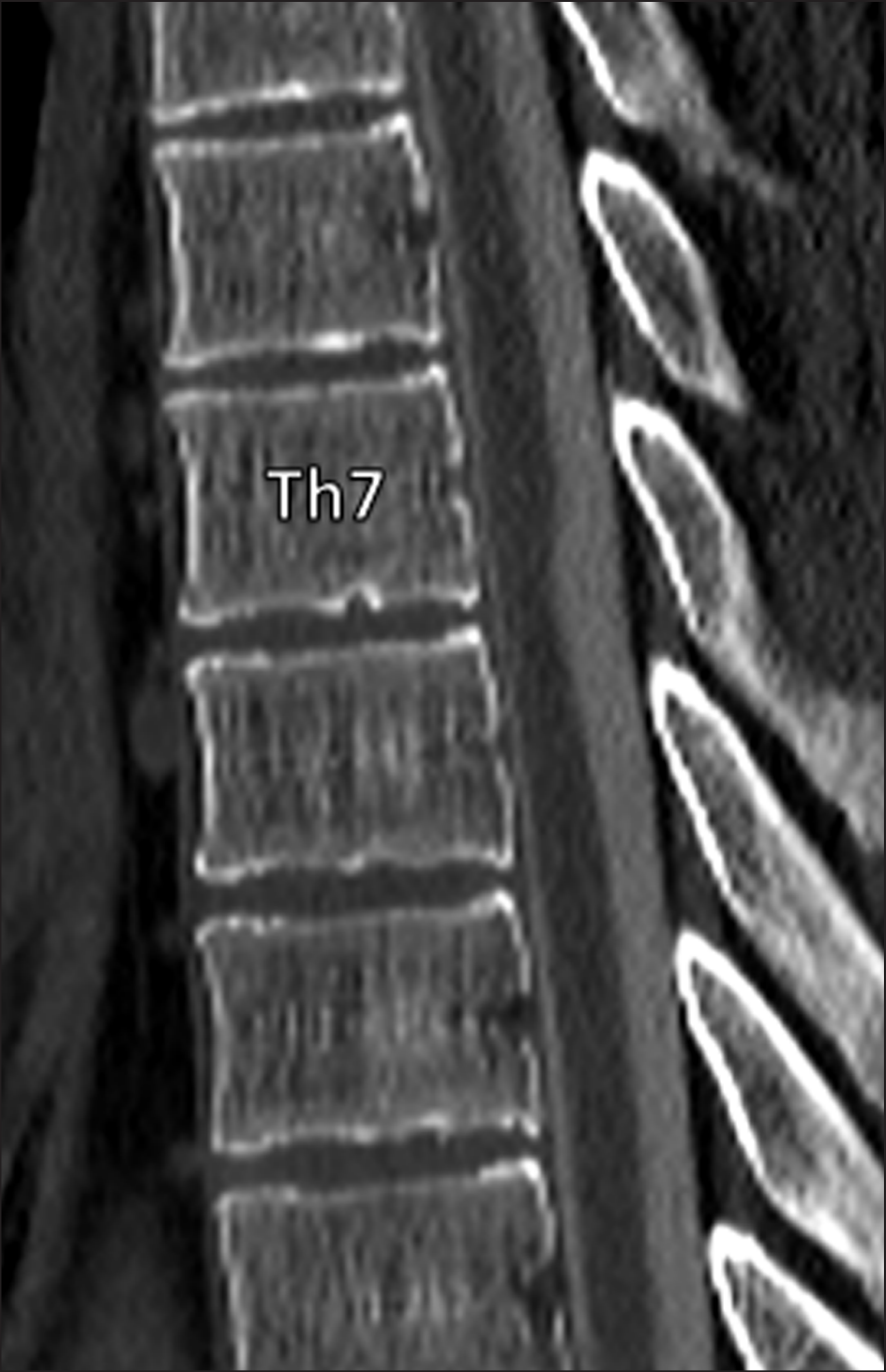


Magnetic resonance imaging (MRI) revealed hydromyelia extending from C5 to Th7 and myelopathy at Th7 (***Figure 1***). Focal narrowing of the spinal cord with ventral displacement and dorsal indentation was noted at Th7. Flow-voids were seen in the dorsal subarachnoid spaces, ruling out a spinal arachnoid cyst. Computed tomography (CT)-myelography showed contrast-filled subarachnoid spaces ventral to the spinal cord, excluding a ventral spinal cord herniation (***Figure 2***).

The imaging findings were suggestive of a dorsal arachnoid web (DAW) causing a typical dorsal indentation of the spinal cord, an imaging finding also referred to as “the scalpel sign”.

**Figure 3:**
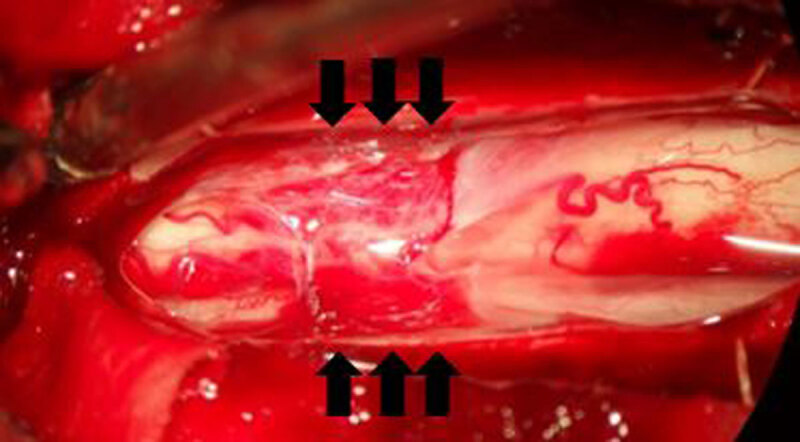


Due to limited response and side effects of conservative treatment, surgery was performed. Perioperative images after opening of the posterior dural sac reveal the presence of a DAW with compression of the spinal cord (***Figure 3***, arrows). The DAW was resected and consisted pathologically of connective tissue lined by meningothelial cells.

The pain and sensory deficits resolved completely, and control MRI showed resolution of the spinal cord deformity and improvement of the spinal cord signal changes.

## Commentary

DAWs are intradural thickened bands of arachnoid tissue at the dorsal aspect of the spinal cord. They have a predilection for the upper thoracic spine and tend to cause a focal dorsal indentation on the spinal cord, resulting in the scalpel sign. It is a rare entity although its true incidence may be underestimated [1].

DAWs are presumed to be incomplete or collapsed arachnoid cysts. Spinal arachnoid cysts are thought to arise from diverticula of the septum posticum, which explains their typical dorsal location. Some reports suggest a history of trauma to be involved in the pathophysiology of DAWs. However, prior trauma is absent in many cases. Other theories include arachnoid herniation in congenital dural defects of post-infectious or post-operative etiologies [1].

The clinical features consist of neuropathic back pain and compressive myelopathy or radiculopathy. DAWs have been reported without neurological deficits, however, and the incidence of asymptomatic DAWs is probably underestimated [1].

MRI is considered the gold standard for investigation of DAWs with its pathognomonic scalpel sign. Although the web generally escapes the spatial resolution of imaging, CISS sequence has been used to directly visualize the web [1]. When symptomatic, DAWs can be associated with hydromyelia and/or spinal cord signal changes.

Resection or fenestration of DAWs is usually curative with regression of the scalpel sign and possible medullary signal changes [1]. Our case revealed some residual spinal cord signal changes after surgery, probably reflecting permanent tissue damage due to chronic myelopathy.

## References

[1] Ben AH, Hamilton P, Zygmunt S, Yakoub KM. Spinal arachnoid web-a review article. J Spine Surg. 2018; 4(2): 446–50. DOI: 10.21037/jss.2018.05.08 30069540 PMC6046336

